# Pharmacological prolyl hydroxylase inhibition enhances high‐intensity exercise capacity in mice

**DOI:** 10.14814/phy2.70464

**Published:** 2025-07-14

**Authors:** Koji Takemura, Motoki Odawara, Hiroshi Nishi, Takaaki Higashihara, Yoko Yoshida, Koichi Nakazato, Nobuharu L. Fujii, Naokata Ishii, Reiko Inagi, Tetsuhiro Tanaka, Masaomi Nangaku

**Affiliations:** ^1^ Division of Nephrology and Endocrinology The University of Tokyo Graduate School of Medicine Tokyo Japan; ^2^ Department of Exercise Physiology Nippon Sport Science University Tokyo Japan; ^3^ Department of Health Promotion Sciences Graduate School of Human Health Sciences, Tokyo Metropolitan University Tokyo Japan; ^4^ Department of Life Sciences The University of Tokyo Graduate School of Arts and Sciences Tokyo Japan; ^5^ Division of CKD Pathophysiology The University of Tokyo Graduate School of Medicine Tokyo Japan

**Keywords:** anemia, exercise capacity, hemoglobin, skeletal muscle

## Abstract

Increased blood hemoglobin concentration theoretically promotes oxygen delivery to the periphery. Clinically, in addition to recombinant human erythropoietin analogs, prolyl hydroxylase (PH) inhibitors to stabilize the expression of hypoxia‐inducible factor (HIF) protein were recently approved for the treatment of anemia in chronic kidney disease. However, whether the new agent helps enhance physical exercise capacity remains a matter of controversy. Treatment of C57BL/6J mice via oral gavage with roxadustat of 30 mg/kg three times per week for 5 weeks elevated the blood hemoglobin concentration, upregulated the HIF‐downstream gene expression of the muscle, and enhanced a high‐intensity exercise performance measured with a treadmill running exhaustion test, compared to those treated with vehicle, while total body weight or skeletal muscle mass was comparable. This physical effect depended on the increase in blood hemoglobin concentration, as confirmed by mice hemodiluted to control hemoglobin concentrations through phlebotomy followed by infusion of the approximate volume of phosphate‐buffered saline. In conclusion, oral administration of the HIF‐PH inhibitor to mice increased high‐intensity exercise capacity as well as elevated blood hemoglobin concentration. The finding implies the versatile effects on humans taking HIF‐PH inhibitors, as well as the risk of them being abused for blood doping.

## 1 INTRODUCTION

Oxygen is a requisite for skeletal muscle function in aerobic conditions. Arterial oxygen content (CaO_2_) is determined by hemoglobin concentration (Hb, g/L), arterial oxygen tension (PaO_2_, mmHg), and arterial oxygen saturation (SaO_2_) as per the equation: CaO_2_ (mL/L) = (SaO_2_ × Hb [g/L] × 1.37) + (PaO_2_ [mmHg] × 0.003). In this regard, the elevation of blood Hb concentration can be beneficial to exercise capacity as oxygen consumption is a major performance‐limiting factor (Bassett Jr. & Howley, 2000).

Cellular responses to changes in oxygen concentration are mediated by post‐translational oxidation of hypoxia‐inducible transcription factors (HIFs). Hydroxylation of prolyl residues within the HIF‐α subunit, catalyzed by HIF prolyl hydroxylase (PH), signals its proteasomal degradation (Maxwell & Eckardt, 2016; Semenza, 2001). Among at least three PH isoforms in mammals, PHD2 is the main isoform responsible for regulating HIF degradation (Berra et al., 2003). Intriguingly, the effect of running training is enhanced in mice deficient in PHD2 (Nunomiya et al., 2017).

Pharmacological inhibitors of HIF‐PH have been developed and are currently approved in the clinical setting to treat anemia in chronic kidney disease (Babitt & Lin, 2012; Odawara et al., 2024). However, in contrast to the phenotype of the mice deficient in PHD2, it so far remains controversial whether HIF‐PH inhibitors exert any ergogenic effect (Beuck et al., 2012; Heuberger et al., 2017; Pottgiesser & Schumacher, 2013). Therefore, we examined the effect of roxadustat, one of these drugs, on the blood Hb concentration and exercise capacity in mice.

## 2 METHODS

### 2.1 Animal experiment

Eight‐week‐old male and female C57BL/6J mice (Clea Japan, Japan) were fed regular chow (#MF, Oriental Yeast, Japan) ad libitum and maintained on a 12‐h light cycle and a 12‐h dark cycle. Mice were administered via oral gavage roxadustat (#S1007, Selleck Chemicals, USA) at 30 mg/kg with a vehicle solution containing 0.5% carboxymethyl cellulose (#C0603, Tokyo Chemical Industry, Japan) with 0.1% polysorbate 80 (#164–21,591, Wako Pure Chemical, Japan) or a vehicle alone three times a week unless otherwise described. During the period, body weight and diet intake were monitored every 2 weeks.

Blood was drawn from the mice at indicated time points. Blood Hb concentration in anticoagulant‐mixed blood samples was measured using an automated blood cell counter, PCE‐210N (Erma, Japan). Plasma erythropoietin concentration was measured with ELISA (#KE10031, Proteintech, USA).

The animals were euthanized at the indicated time points for further analysis. The kidney, liver, and skeletal muscle tissues were harvested on day 0, 14, or 35. Mice used for the phlebotomy experiment on day 35 were treated with roxadustat until day 42.

### 2.2 Immunofluorescence histochemical staining analysis

Immunofluorescence histochemical staining analysis was performed as previously described with modification (Higashihara et al., 2021, 2024). In brief, the gastrocnemius (GC) muscles were harvested from the mice, embedded in the Tissue‐Teck O.C.T compound (#4583, Sakura Finetek, Japan), and snap frozen in isopentane cooled by liquid nitrogen. The cryosections of 5 μm‐thick slices were blocked in blocking solution (#X0909, Dako, Denmark) for 10 min at room temperature. Slices were then incubated overnight at 4°C with primary antibodies, such as the anti‐laminin antibody (1:500, #AB19012, Sigma‐Aldrich, USA) or anti‐slow myosin (1:1000, #M8421, Sigma‐Aldrich, USA) antibodies in phosphate‐buffered saline containing 1% bovine serum albumin. Section slices were washed in phosphate‐buffered saline, and then incubated with secondary antibodies such as Alexa Fluor 488 anti‐mouse IgG antibody (1:2000, #AB150113, Abcam, United Kingdom) and Alexa Fluor 594 anti‐rabbit IgG antibody (1:300, #A21207, Thermo Fisher Scientific, USA) for 1 h at room temperature. Image acquisition was performed with an inverted fluorescence microscope, BZ‐X710 (Keyence, Japan). Slow myosin‐positive area was standardized by the whole area of the section in each sample using ImageJ software version 1.54d (NIH, USA).

### 2.3 Hemodilution procedure

Hemodilution was conducted by phlebotomy followed by injection of an equal volume of phosphate‐buffered saline as previously described with modification (Eto et al., 2007). In brief, the proportion of the drawn volume to the total circulating volume was determined by comparison of the average blood Hb concentration between two groups, and the calculated proportion was applied to all mice treated with roxadustat. The total circulating blood volume was estimated as 72 mL/kg with reference to a previously published practice guide (Diehl et al., 2001). A sham procedure was executed on the control group by just anesthesia and pricking.

### 2.4 Reverse transcription quantitative PCR

Mouse tissue RNA was isolated with RNAiso Plus (#9108, Takara, Japan) and reverse transcribed to cDNA using PrimeScript RT master mix (#RR036A, Takara, Japan). Quantitative PCR was performed on a CFX96 cycler CFX96 System (Bio‐Rad Laboratories, USA) with THUNDERBIRD SYBR (#QPS‐201, Toyobo, Japan). The primers used (Fasmac, Japan) were described in Table 1.

**TABLE 1:** Primers used for RT‐PCR in this study.

Gene	Species		Sequence
*Pdk1*	Mouse	Forward	GGGCCAGGTGGACTTCTATG
		Reverse	TGGATATACCAACTTTGCACCAG
*Glut1*	Mouse	Forward	GTGACGATCTGAGCTACGGG
		Reverse	ACTCCTCAATAACCTTCTGGGG
*Vegf*	Mouse	Forward	TTACTGCTGTACCTCCACCA
		Reverse	ACAGGACGGCTTGAAGATG
*Hmox1*	Mouse	Forward	GCCACCAAGGAGGTACACAT
		Reverse	GCTTGTTGCGCTCTATCTCC
*Actb*	Mouse	Forward	AAGATCAAGATCATTGCTCCTCCTG
		Reverse	AAACGCAGCTCAGTAACAGTCC

### 2.5 Treadmill running exhaustion test

The treadmill exhaustion test to evaluate a high‐intensity exercise capacity was conducted using the mouse treadmill device, TMS‐8B (Melquest, Japan), which was set up horizontally. Mice ran individually on separate lanes with shock‐grids (stimulatory shock of 0.3 mA) at the rear end. After acclimation to the treadmill at a speed slower than 10 m/min, treadmill tests started at a speed of 10 m/min and then the treadmill velocity gradually increased by 3 m/min every 2 min. When the mice remained in the shock‐grid area for more than 5 s, they were regarded as exhausted and were promptly removed from the lanes. The maximum velocity (Vmax) and running distance and time at the dropout were recorded.

### 2.6 Statistical analysis

Statistical analysis was performed with a two‐tailed, unpaired or paired Student's *t*‐test, or calculation of Pearson's correlation coefficient as indicated. A *p* value less than 0.05 was considered statistically significant.

## 3 RESULTS

### 3.1 One‐time orally administered roxadustat upregulated the expression of HIF‐downstream genes in multiple organs and sites

**FIGURE 1:**
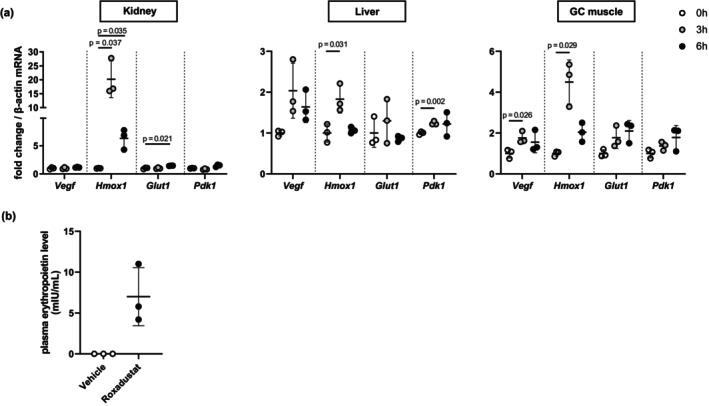
One‐time roxadustat treatment increased HIF‐downstream gene expression in liver, kidney, and skeletal muscle and elevated plasma erythropoietin concentration. (a) Reverse transcription quantitative PCR analysis was conducted to evaluate mRNA expression of HIF‐targeted genes in the kidney, the liver, and the gastrocnemius (GC) muscles at 0 h, 3 h, and 6 h after 30 mg/kg roxadustat or vehicle administration (*n* = 3 mice per group). (b) Plasma erythropoietin concentrations at 6 h after 30 mg/kg roxadustat or vehicle administration to normal mice were shown (*n* = 3 mice per group). Bars indicate mean values ± SD.

First, we evaluated the drug delivery and effect in multiple organs and sites after administration of roxadustat. When the reagent was administered via oral gavage to mice, the expression of HIF‐1 downstream genes including *Hmox1* was increased in the liver and the kidney (Figure 1a). Further, the expression of HIF‐1 downstream genes such as *Hmox1* and *Vegf* was increased in the GC muscle (Figure 1a). Similarly, the plasma erythropoietin concentration was elevated from zero by the treatment (Figure 1b).

### 3.2 Five‐week administration of roxadustat enhanced a high‐intensity exercise capacity of mice with the increase in blood Hb concentration

**FIGURE 2:**
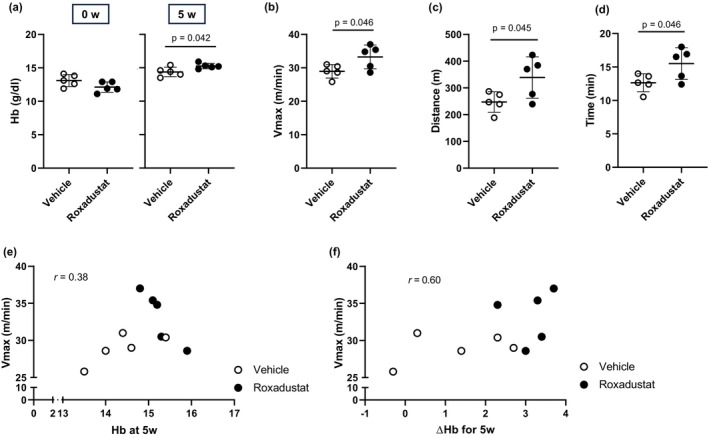
Five‐week roxadustat treatment elevated blood hemoglobin concentration and increased a high‐intensity exercise capacity. In mice treated with 30 mg/kg roxadustat or vehicle via oral gavage three times per week for 5 weeks, (a) blood hemoglobin (Hb) concentration was measured. Then, (b) Vmax, (c) total running distance, and (d) time of the mice forced to run to exhaustion over a conveyor belt with gradually increasing speed are shown. Correlation of Vmax to (e) blood Hb concentration at 5 weeks and (f) the increase in the Hb concentration (ΔHb) for 5 weeks is expressed as dot plots (*n* = 5 mice per group). Bars indicate mean values ± SD.

While oral gavage administration of roxadustat or vehicle was repeated three times per week, the blood Hb concentration, physical activity, or skeletal muscle weight did not differ between the groups at 2 weeks (data not shown). However, at 5 weeks, mice treated with roxadustat displayed a mild elevation in blood Hb concentrations compared to that in mice treated with vehicle (Figure 2a), which agrees with previous findings (Beck et al., 2017). Further, mice treated with roxadustat showed greater exercise capacity on a treadmill running exhaustion test compared to control mice (Figure 2b–d). The Hb concentration at 5 weeks (Figure 2e, *r* = 0.38) or the increase in the Hb concentration for 5 weeks (Figure 2f, *r* = 0.60) were positively correlated with the maximal velocity recorded in the treadmill test, all suggesting an association of blood Hb concentration with a high‐intensity exercise capacity.

**FIGURE 3:**
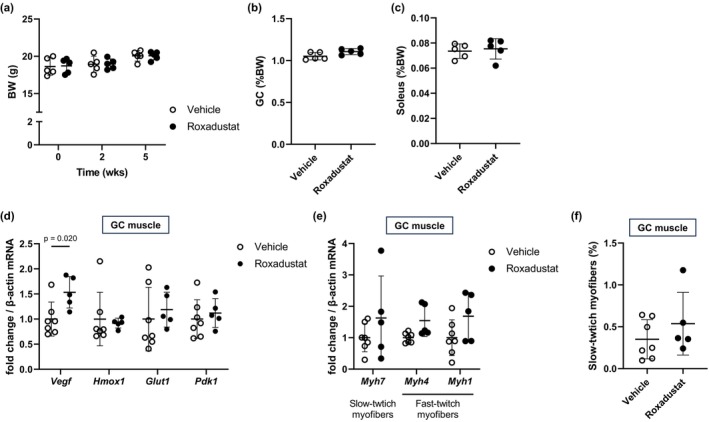
Five‐week roxadustat treatment did not change muscle mass or induce myofiber type switch. In mice treated with 30 mg/kg roxadustat or vehicle via oral gavage three times per week for 5 weeks, (a) total body weight (BW) and wet mass of (b) the gastrocnemius (GC) and (c) the soleus muscle from bilateral hindlimbs is shown as % total body weight (*n* = 5 mice per group). (d, e) Reverse transcription quantitative PCR analysis was conducted to evaluate mRNA expression of HIF‐targeted genes in the GC muscle at 6 h after the last administration of 30 mg/kg roxadustat or vehicle (*n* = 5–7 mice per group). (f) Frozen sections of GC muscles were stained for slow‐twitch myofibers by fluorescent immunohistochemistry using an anti‐laminin antibody and an anti‐slow myosin antibody along with secondary antibodies, and the area of the stained region was expressed as a percentage of the total cross‐sectional area of muscle tissue (*n* = 5–7 mice per group). Bars indicate mean values ± SD.

Next, we explored the mechanism to underlie this difference in the exercise capacity. At least, no difference was observed in the weight of the whole body (Figure 3a), the GC muscle (Figure 3b), and the soleus (Figure 3c). Notably, *Vegf*, one of the HIF‐target genes, expression of the GC muscle was increased in the mice treated with roxadustat for 5 weeks compared to the mice treated with vehicle (Figure 3d). Regarding the myofiber typing, the balance of slow‐ or fast‐twitch distribution was not affected by roxadustat treatment in terms of gene expression (Figure 3e) or histology (Figure 3f).

### 3.3 Forced reduction of blood Hb concentration via phlebotomy attenuated the ergogenic effect caused by 5‐week administration of roxadustat

**FIGURE 4:**
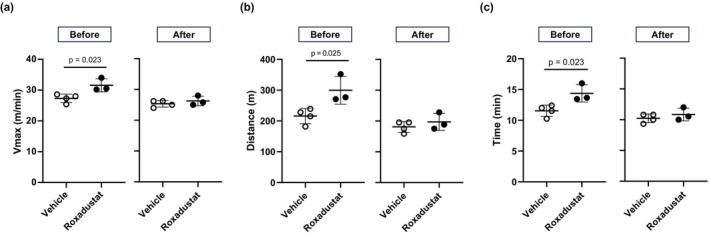
Increase in a high‐intensity exercise capacity by 5‐week roxadustat treatment was attenuated by hemodilution procedure. In mice after 5‐week roxadustat treatment, the effect of forced reduction of blood Hb concentration on exercise capacity was evaluated. (a) Vmax, (b) total running distance, and (c) time in treadmill running exhaustion test before and after hemodilution is shown (*n* = 3–4 mice per group). Bars indicate mean values ± SD.

By contract, to assess the effect of elevated blood Hb concentration and presumably, subsequent oxygen delivery, on physical performance, we conducted phlebotomy followed by injecting an equal volume of phosphate‐buffered saline based on an arithmetic estimation of the appropriate blood volume drawn. This experimental procedure blunted the reinforcement of exercise capacity (Figure 4a–c), suggesting that roxadustat increased exercise capacity mainly by the elevation of blood Hb concentration.

## 4 DISCUSSION

In this study, we showed that repeated roxadustat treatment increased high‐intensity exercise capacity in mice, which depends on the pharmacological HIF‐PH inhibition‐induced increase in blood Hb concentration.

A high‐intensity exercise performance is determined by skeletal uptake and utilization of oxygen, which are limited by the cardiorespiratory system's ability to deliver oxygen to the end organs (Bassett Jr. & Howley, 2000). Regarding the effect of blood doping in elite athletes, however, the presumably propitious role of erythropoietic agents to enhance physical performance is controversial (Heuberger et al., 2017). Our results suggest that the physical performance capacity could be positively affected by HIF‐PH inhibitor–mediated elevation of the blood Hb concentration. As erythropoietin and its analogues as well as HIF‐PH inhibitors are abused by athletes as detected using mass spectrometric analysis (Buisson et al., 2016), our findings warrant the development of a drug detection technology or individual monitoring of blood Hb concentrations as anti‐blood doping tests (Atkinson & Kahn, 2020).

In our results, a linear relationship was not robust between Hb and Vmax. This may be explained by the optimal range of Hb concentrations for exercise performance (Schuler et al., 2010). Roxadustat treatment not only increases blood Hb concentration but also may elevate blood viscosity, negatively affecting cardiac performance (Schuler et al., 2010). Thus, blood viscosity may correlate with maximal oxygen consumption of the skeletal muscles, which are not evaluated in the current study.

During high‐intensity exercise, the skeletal muscle highly demands oxygen and may encounter relative oxygen shortage. Thus, under hypoxic conditions, tissue oxygen consumption is linked to the formation of reactive oxygen species (Kim et al., 2006). Therefore, HIF‐1α stabilization in the skeletal muscle of our model may help resist oxidative stress and exert a positive effect on exercise capacity. Indeed, HIF‐1 ablation selectively in skeletal muscle causes tissue damage to skeletal muscle after repetitive exercise and deteriorated performance, such as swimming or running time (Mason et al., 2004). Notably, the conditional mutant mice show no elevation of blood Hb concentration (Mason et al., 2004), probably since HIF‐2 but not HIF‐1 is more crucial in controlling erythropoietin synthesis (Haase, 2013). In addition, in humans, training exercise modulates the activity of HIFs in the skeletal muscle of athletes (Lindholm & Rundqvist, 2016). These findings suggest that HIF‐1α is essential to control the performance of skeletal muscle.

Although the forced reduction in blood Hb concentration by phlebotomy attenuated the muscle strength‐enhancing effect of HIF‐PH inhibitors, possible effects other than erythropoiesis include slow‐to‐fast type switch in skeletal muscle fibers (Pisani & Dechesne, 2005; Rasbach et al., 2010), angiogenesis and proliferation in muscle tissue (Moeller et al., 2004), and enhanced glucose transportation (Fujii et al., 2006) and anaerobic glycolysis (Lu et al., 2002), all of which could be caused by HIF protein stabilization. Although downregulation was not observed in slow muscle myofiber proportions in our analysis, other factors have not been fully examined and thus need further investigation.

In the clinical practice, HIF‐PH inhibitors have been approved for the treatment of anemia in chronic kidney disease. The deterioration of physical activity in those patients is multifaceted (Duarte et al., 2024; Nishi et al., 2020; Takemura et al., 2020), including the decline of cognitive function and motivation, and muscle function (Higashihara et al., 2024). Our findings indicate that this drug may be favorable at least to the decreased exercise performance among those patients, as shown in mutant mice that lack PHD2 selectively in the skeletal muscles (Nunomiya et al., 2017). However, this probably requires long‐term treatment (at least for several weeks) with these drugs, which may exert potentially unfavorable effects (Barratt et al., 2021; Haraguchi et al., 2023). In addition, patients suffering from chronic kidney disease are usually elderly and frail (Nixon et al., 2018), and therefore may not be fully relevant to the high‐intensity performance that is the outcome of this study. Also, in the actual settings, clinical consensus on the treatment of anemia in kidney diseases is based on the studies targeting patients in whom conventional erythropoiesis stimulating agents were used (Ku et al., 2023), so whether the same goals and treatment standards should be used for anemia treatment with HIF‐PH inhibitors will need to be discussed in the future.

In summary, HIF‐PH inhibitors could enhance high‐intensity exercise capacity through increasing blood Hb concentrations in mice, suggesting that HIF‐PH inhibitors may have propitious effects in patients undergoing this treatment, and may even be inappropriately used in athletes as a means of blood doping. Future studies are needed to determine whether the ergogenic effects of HIF‐PH inhibitors are observed in humans.

## AUTHOR CONTRIBUTIONS

K.T., H.N., and M.N. conceived the study. K.T., M.O., and H.N. designed the experiments, analyzed the data, and wrote the original manuscript. K.T., M.O., and Y.Y. performed the experiments. T.H. and K.N. provided essential technical advice. H.N., Y.Y., N.F.L., N.I., R.I., T.T., and M.N. provided conceptual advice and edited the manuscript.

## FUNDING INFORMATION

This work was supported by MEXT/JSPS Grant‐in‐Aids 24 K11425 (to H.N.), 21H02824 (to R.I.), 23 K07713 (to T.T.), 23 K27615 (M.N.) and by the Nakatomi Foundation, the Mitsui Sumitomo Insurance Welfare Foundation, the Descente and Ishimoto Memorial Foundation for the Promotion of Sports Science, and the Suzuken Memorial Foundation (to H.N.).

## CONFLICT OF INTEREST STATEMENT

H.N. reported receiving honoraria from Astellas, AstraZeneca, MSD, Otsuka, Ono, Kyowa Kirin, Kowa, Daiichi Sankyo, Mitsubishi Tanabe, Torii, Eli Lilly, Boehringer Ingelheim, and Bayer; and research grants from Mitsubishi Tanabe, Boehringer Ingelheim, Novo Nordisk, Bayer, and Kyowa Kirin. M.N. reported receiving research funding from Kyowa Kirin, Daiichi Sankyo, Astellas, Ono, Mitsubishi Tanabe, JT, Chugai, Bayer, Torii, and Takeda; and reported receiving honoraria and/or advisory fees from Kyowa Kirin, Astellas, AstraZeneca, GSK, Daiichi Sankyo, Mitsubishi Tanabe, Chugai, Torii, JT, Novo Nordisk, and Boehringer Ingelheim. All remaining authors have nothing to disclose.

## ETHICS STATEMENT

All animal experiments were conducted in accordance with the guidelines for the care and use of laboratory animals, and were approved by the Faculty of Medicine, University of Tokyo Institutional Animal Care and Use Committee (M‐P19‐003).

## Data Availability

Data are available on request from authors.

## References

[phy270464-bib-0001] Atkinson, T. S. , & Kahn, M. J. (2020). Blood doping: Then and now. A narrative review of the history, science and efficacy of blood doping in elite sport. Blood Reviews, 39, 100632. 31645265 10.1016/j.blre.2019.100632

[phy270464-bib-0002] Babitt, J. L. , & Lin, H. Y. (2012). Mechanisms of anemia in CKD. Journal of the American Society of Nephrology, 23, 1631–1634. 22935483 10.1681/ASN.2011111078 PMC3458456

[phy270464-bib-0003] Barratt, J. , Andric, B. , Tataradze, A. , Schomig, M. , Reusch, M. , Valluri, U. , & Mariat, C. (2021). Roxadustat for the treatment of anaemia in chronic kidney disease patients not on dialysis: A phase 3, randomized, open‐label, active‐controlled study (DOLOMITES). Nephrology, Dialysis, Transplantation, 36, 1616–1628. 10.1093/ndt/gfab191 PMC8396401 34077510

[phy270464-bib-0004] Bassett, D. R., Jr. , & Howley, E. T. (2000). Limiting factors for maximum oxygen uptake and determinants of endurance performance. Medicine and Science in Sports and Exercise, 32, 70–84. 10647532 10.1097/00005768-200001000-00012

[phy270464-bib-0005] Beck, J. , Henschel, C. , Chou, J. , Lin, A. , & Del Balzo, U. (2017). Evaluation of the carcinogenic potential of Roxadustat (FG‐4592), a small molecule inhibitor of hypoxia‐inducible factor prolyl hydroxylase in CD‐1 mice and Sprague Dawley rats. International Journal of Toxicology, 36, 427–439. 29153032 10.1177/1091581817737232

[phy270464-bib-0006] Berra, E. , Benizri, E. , Ginouves, A. , Volmat, V. , Roux, D. , & Pouyssegur, J. (2003). HIF prolyl‐hydroxylase 2 is the key oxygen sensor setting low steady‐state levels of HIF‐1alpha in normoxia. The EMBO Journal, 22, 4082–4090. 12912907 10.1093/emboj/cdg392 PMC175782

[phy270464-bib-0007] Beuck, S. , Schanzer, W. , & Thevis, M. (2012). Hypoxia‐inducible factor stabilizers and other small‐molecule erythropoiesis‐stimulating agents in current and preventive doping analysis. Drug Testing and Analysis, 4, 830–845. 22362605 10.1002/dta.390

[phy270464-bib-0008] Buisson, C. , Marchand, A. , Bailloux, I. , Lahaussois, A. , Martin, L. , & Molina, A. (2016). Detection by LC–MS/MS of HIF stabilizer FG‐4592 used as a new doping agent: Investigation on a positive case. Journal of Pharmaceutical and Biomedical Analysis, 121, 181–187. 26808067 10.1016/j.jpba.2016.01.029

[phy270464-bib-0009] Diehl, K. H. , Hull, R. , Morton, D. , Pfister, R. , Rabemampianina, Y. , Smith, D. , Vidal, J. M. , van de Vorstenbosch, C. , European Federation of Pharmaceutical Industries a , & European Centre for the Validation of alternative M . (2001). A good practice guide to the administration of substances and removal of blood, including routes and volumes. Journal of Applied Toxicology, 21, 15–23. 11180276 10.1002/jat.727

[phy270464-bib-0010] Duarte, M. P. , Almeida, L. S. , Neri, S. G. R. , Oliveira, J. S. , Wilkinson, T. J. , Ribeiro, H. S. , & Lima, R. M. (2024). Prevalence of sarcopenia in patients with chronic kidney disease: A global systematic review and meta‐analysis. Journal of Cachexia, Sarcopenia and Muscle, 15, 501–512. 38263952 10.1002/jcsm.13425 PMC10995263

[phy270464-bib-0011] Eto, N. , Wada, T. , Inagi, R. , Takano, H. , Shimizu, A. , Kato, H. , Kurihara, H. , Kawachi, H. , Shankland, S. J. , Fujita, T. , & Nangaku, M. (2007). Podocyte protection by darbepoetin: Preservation of the cytoskeleton and nephrin expression. Kidney International, 72, 455–463. 17457371 10.1038/sj.ki.5002311

[phy270464-bib-0012] Fujii, N. , Jessen, N. , & Goodyear, L. J. (2006). AMP‐activated protein kinase and the regulation of glucose transport. American Journal of Physiology. Endocrinology and Metabolism, 291, E867–E877. 16822958 10.1152/ajpendo.00207.2006

[phy270464-bib-0013] Haase, V. H. (2013). Regulation of erythropoiesis by hypoxia‐inducible factors. Blood Reviews, 27, 41–53. 23291219 10.1016/j.blre.2012.12.003 PMC3731139

[phy270464-bib-0014] Haraguchi, T. , Hamamoto, Y. , Kuwata, H. , Yamazaki, Y. , Nakatani, S. , Hyo, T. , Yamada, Y. , Yabe, D. , & Seino, Y. (2023). Effect of Roxadustat on thyroid function in patients with renal anemia. The Journal of Clinical Endocrinology and Metabolism, 109, e69–e75. 37597171 10.1210/clinem/dgad483

[phy270464-bib-0015] Heuberger, J. , Rotmans, J. I. , Gal, P. , Stuurman, F. E. , van't Westende, J. , Post, T. E. , Daniels, J. M. A. , Moerland, M. , van Veldhoven, P. L. J. , de Kam, M. L. , Ram, H. , de Hon, O. , Posthuma, J. J. , Burggraaf, J. , & Cohen, A. F. (2017). Effects of erythropoietin on cycling performance of well trained cyclists: A double‐blind, randomised, placebo‐controlled trial. The Lancet Haematology, 4, e374–e386. 28669689 10.1016/S2352-3026(17)30105-9

[phy270464-bib-0016] Higashihara, T. , Nishi, H. , Takemura, K. , Watanabe, H. , Maruyama, T. , Inagi, R. , Tanaka, T. , & Nangaku, M. (2021). beta2‐adrenergic receptor agonist counteracts skeletal muscle atrophy and oxidative stress in uremic mice. Scientific Reports, 11, 9130. 33911115 10.1038/s41598-021-88438-7 PMC8080640

[phy270464-bib-0017] Higashihara, T. , Odawara, M. , Nishi, H. , Sugasawa, T. , Suzuki, Y. , Kametaka, S. , Inagi, R. , & Nangaku, M. (2024). Uremia impedes skeletal myocyte Myomixer expression and Fusogenic activity: Implication for uremic sarcopenia. The American Journal of Pathology, 194, 759–771. 38637109 10.1016/j.ajpath.2024.01.005

[phy270464-bib-0018] Kim, J. W. , Tchernyshyov, I. , Semenza, G. L. , & Dang, C. V. (2006). HIF‐1‐mediated expression of pyruvate dehydrogenase kinase: A metabolic switch required for cellular adaptation to hypoxia. Cell Metabolism, 3, 177–185. 16517405 10.1016/j.cmet.2006.02.002

[phy270464-bib-0019] Ku, E. , Del Vecchio, L. , Eckardt, K. U. , Haase, V. H. , Johansen, K. L. , Nangaku, M. , Tangri, N. , Waikar, S. S. , Wiecek, A. , Cheung, M. , Jadoul, M. , Winkelmayer, W. C. , Wheeler, D. C. , & for Conference P . (2023). Novel anemia therapies in chronic kidney disease: Conclusions from a kidney disease: Improving global outcomes (KDIGO) controversies Conference. Kidney International, 104, 655–680. 37236424 10.1016/j.kint.2023.05.009

[phy270464-bib-0020] Lindholm, M. E. , & Rundqvist, H. (2016). Skeletal muscle hypoxia‐inducible factor‐1 and exercise. Experimental Physiology, 101, 28–32. 26391197 10.1113/EP085318

[phy270464-bib-0021] Lu, H. , Forbes, R. A. , & Verma, A. (2002). Hypoxia‐inducible factor 1 activation by aerobic glycolysis implicates the Warburg effect in carcinogenesis. The Journal of Biological Chemistry, 277, 23111–23115. 11943784 10.1074/jbc.M202487200

[phy270464-bib-0022] Mason, S. D. , Howlett, R. A. , Kim, M. J. , Olfert, I. M. , Hogan, M. C. , McNulty, W. , Hickey, R. P. , Wagner, P. D. , Kahn, C. R. , Giordano, F. J. , & Johnson, R. S. (2004). Loss of skeletal muscle HIF‐1alpha results in altered exercise endurance. PLoS Biology, 2, e288. 15328538 10.1371/journal.pbio.0020288 PMC514537

[phy270464-bib-0023] Maxwell, P. H. , & Eckardt, K. U. (2016). HIF prolyl hydroxylase inhibitors for the treatment of renal anaemia and beyond. Nature Reviews. Nephrology, 12, 157–168. 26656456 10.1038/nrneph.2015.193

[phy270464-bib-0024] Moeller, B. J. , Cao, Y. , Vujaskovic, Z. , Li, C. Y. , Haroon, Z. A. , & Dewhirst, M. W. (2004). The relationship between hypoxia and angiogenesis. Seminars in Radiation Oncology, 14, 215–221. 15254864 10.1016/j.semradonc.2004.04.005

[phy270464-bib-0025] Nishi, H. , Takemura, K. , Higashihara, T. , & Inagi, R. (2020). Uremic sarcopenia: Clinical evidence and basic experimental approach. Nutrients, 12, 1814. 32570738 10.3390/nu12061814 PMC7353433

[phy270464-bib-0026] Nixon, A. C. , Bampouras, T. M. , Pendleton, N. , Woywodt, A. , Mitra, S. , & Dhaygude, A. (2018). Frailty and chronic kidney disease: Current evidence and continuing uncertainties. Clinical Kidney Journal, 11, 236–245. 29644065 10.1093/ckj/sfx134 PMC5888002

[phy270464-bib-0027] Nunomiya, A. , Shin, J. , Kitajima, Y. , Dan, T. , Miyata, T. , & Nagatomi, R. (2017). Activation of the hypoxia‐inducible factor pathway induced by prolyl hydroxylase domain 2 deficiency enhances the effect of running training in mice. Acta Physiologica (Oxford, England), 220, 99–112. 27393382 10.1111/apha.12751 PMC5412909

[phy270464-bib-0028] Odawara, M. , Nishi, H. , & Nangaku, M. (2024). A spotlight on using HIF‐PH inhibitors in renal anemia. Expert Opinion on Pharmacotherapy, 25, 1291–1299. 38994698 10.1080/14656566.2024.2378903

[phy270464-bib-0029] Pisani, D. F. , & Dechesne, C. A. (2005). Skeletal muscle HIF‐1alpha expression is dependent on muscle fiber type. The Journal of General Physiology, 126, 173–178. 16043777 10.1085/jgp.200509265 PMC2266573

[phy270464-bib-0030] Pottgiesser, T. , & Schumacher, Y. O. (2013). Current strategies of blood doping detection. Analytical and Bioanalytical Chemistry, 405, 9625–9639. 23934350 10.1007/s00216-013-7270-x

[phy270464-bib-0031] Rasbach, K. A. , Gupta, R. K. , Ruas, J. L. , Wu, J. , Naseri, E. , Estall, J. L. , & Spiegelman, B. M. (2010). PGC‐1alpha regulates a HIF2alpha‐dependent switch in skeletal muscle fiber types. Proceedings of the National Academy of Sciences of the United States of America, 107, 21866–21871. 21106753 10.1073/pnas.1016089107 PMC3003089

[phy270464-bib-0032] Schuler, B. , Arras, M. , Keller, S. , Rettich, A. , Lundby, C. , Vogel, J. , & Gassmann, M. (2010). Optimal hematocrit for maximal exercise performance in acute and chronic erythropoietin‐treated mice. Proceedings of the National Academy of Sciences of the United States of America, 107, 419–423. 19966291 10.1073/pnas.0912924107 PMC2806754

[phy270464-bib-0033] Semenza, G. L. (2001). HIF‐1, O(2), and the 3 PHDs: How animal cells signal hypoxia to the nucleus. Cell, 107, 1–3. 11595178 10.1016/s0092-8674(01)00518-9

[phy270464-bib-0034] Takemura, K. , Nishi, H. , & Inagi, R. (2020). Mitochondrial dysfunction in kidney disease and uremic sarcopenia. Frontiers in Physiology, 11, 565023. 33013483 10.3389/fphys.2020.565023 PMC7500155

